# Retraction: Biochanin A Gastroprotective Effects in Ethanol-Induced Gastric Mucosal Ulceration in Rats

**DOI:** 10.1371/journal.pone.0294005

**Published:** 2023-11-10

**Authors:** The PLOS ONE Editors

Following the publication of this article [1], concerns were raised regarding reuse of previously published results presented in Figs. 4 and 6. Specifically,

- Fig 4D of this article [1] appears to partially overlap with Fig 8F of [2, retracted in 3]* when rotated, despite being used to represent different experimental conditions.
- Fig 6A of this article [1] appears to partially overlap with the bottom panel in Fig 5B of [4]*, despite being used to represent different experimental conditions.

Given the nature of the issues, the *PLOS ONE* Editors are concerned about the reliability of data management and/or reporting for this study [1].

Following editorial communication regarding these concerns, one of the co-authors requested retraction of the article. The original data underlying this study were not provided for editorial review.

In light of the above concerns, the *PLOS ONE* Editors retract this article.

Some figure panels discussed above appear to report previously published material that are offered under a CC BY license, but the original article(s) was/were not attributed in [1]. For these images, the * by the citation, above, marks the oldest publication of the image of which PLOS is aware.

NAM, HES, and MAA agreed with the retraction. MH responded but expressed neither agreement nor disagreement with the editorial decision. NAS, HK, MZ, KS, RAB, SAMK, and HMA either did not respond directly or could not be reached. NAM and HES apologize for the issues with the published article. MAA stands by the article’s findings.
